# Sympathetic Reinnervation of Intact and Upper Follicle Xenografts into BALB/c-nu/nu Mice

**DOI:** 10.3390/life13112163

**Published:** 2023-11-04

**Authors:** Xiu-Wen Chen, Na Ni, Xiao-Jun Xie, Ying-Lin Zhao, Wen-Zi Liang, Yu-Xin Huang, Chang-Min Lin

**Affiliations:** Department of Histology and Embryology, Shantou University Medical College, Shantou 515041, China; 11xwchen1@stu.edu.cn (X.-W.C.); nani@stu.edu.cn (N.N.); xjxie@stu.edu.cn (X.-J.X.); zyl2001@sina.com (Y.-L.Z.); 20wzliang1@stu.edu.cn (W.-Z.L.); 21yxhuang@stu.edu.cn (Y.-X.H.)

**Keywords:** sympathetic fibers, postganglionic, regeneration, hair follicle, heterograft, denervation

## Abstract

Increasing concerns about hair loss affect people’s quality of life. Recent studies have found that sympathetic nerves play a positive role in regulating hair follicle stem cell activity to promote hair growth. However, no study has investigated sympathetic innervation of transplanted follicles. Rat vibrissa follicles were extracted and implanted under the dorsal skin of BALB/c-nu/nu mice using one of two types of follicles: (1) intact follicles, where transplants included bulbs, and (2) upper follicles, where transplants excluded bulbs. Follicular samples were collected for hematoxylin and eosin staining, immunofluorescence staining for tyrosine hydroxylase (TH, a sympathetic marker) and enzyme-linked immunosorbent assays. At 37 days after implantation in both groups, follicles had entered anagen, with the growth of long hair shafts; tyrosine-hydroxylase-positive nerves were innervating follicles (1.45-fold); and norepinephrine concentrations (2.03-fold) were significantly increased compared to 5 days, but did not return to normal. We demonstrate the survival of intact and upper follicle xenografts and the partial restoration of sympathetic reinnervations of both transplanted follicles.

## 1. Introduction

Hair loss is a common issue worldwide and causes emotional problems, affecting quality of life [1]. Hair transplantation is clinically the only available treatment option for permanent hair loss, and has been used for the treatment of trauma, some types of inflammatory hair diseases, and androgenetic alopecia [2]. Surgeons extract follicular units on the premise that there will be no effect on the appearance of the donor area, and only a limited number of follicular units are taken [3]. 

Hair follicles undergo stages of growth (anagen), involution (catagen) and rest (telogen) [4]. Dermal papilla (DP) cells, a group of specialized fibroblast-like cells located in the hair bulb and at the base of the hair follicle (HF), can efficiently induce follicle regeneration and hair growth [5]. Horne et al. implanted material containing dermal sheath (DS) cells into the remaining upper cavity of the HF and showed histologic restoration of the DP and hair growth [6]. Hashimoto et al. also obtained the same results, achieving hair growth after human HFs with bulb amputation were grafted onto combined severely immunodeficient mice [7]. These results suggest that upper follicular transplantation can double the number of follicles. 

Follicles receive sensory and autonomic (mainly sympathetic) innervation [8,9]. Sensory nerve endings and terminal Schwann cells densely surround the HF central isthmus, forming a lanceolate complex, that is, a neuromuscular-junction-like structure, which enables hair to properly detect hair movement [8,10]. Sympathetic nerves play a positive role in regulating hair follicle stem cell (HFSC) activity to promote hair growth [9,11,12]. Sympathetic nerves form synapse-like connections with Adrb2 (beta-2 adrenergic) receptors on the HFSC surface and use norepinephrine to act as the catecholamine neurotransmitter to initiate several physiological changes, including upregulating canonical cAMP (cyclic adenosine monophosphate)/CRE (cAMP response element)-binding protein signaling to promote glycolytic metabolism, inhibiting the Foxp1-Fgf18 axis and enhancing hedgehog signaling to stimulate HFSCs located in the bulge region of the HF to regenerate follicles in the anagen stage [4,9,11,13]. 

The sympathetic nervous system has a critical role in the regulation of “fight-or-flight” responses after foreign insults and the maintenance of physiological functions, including heart rate and blood pressure under steady state in the cardiovascular system, regulating the chemotaxis of hematopoietic stem and progenitor cells to their niches in hematopoietic system, and inducing islet cell migration and maturation to form the pancreatic islets of Langerhans [14,15]. Many studies used tyrosine hydroxylase (TH) as a sympathetic marker [9,11,16]. Norepinephrine is the primary neurotransmitter in the sympathetic nervous system and its synthesis requires biochemical materials, which must be transported from the ganglion to the nerve terminal via axonal transport. However, sympathetic denervation would deplete the catecholamine stores in the organ [17,18]. Sympathetic denervation can result in organ dysfunction following organ transplantation, where Wallerian degeneration might occur, causing Schwann cells to produce cytokines to recruit monocytes and macrophages to clear the axon debris, and degenerating the myelin of peripheral sympathetic nerves after interruption of the connections between the peripheral and central sympathetic nerves [19]. Subsequent reinnervation occurs, possibly as a result of the intrinsic plasticity of the developing noradrenergic system [20]. Heart transplantation surgically disconnects the postganglionic sympathetic nerve fibers to the myocardium, resulting in cardiac denervation, and some extent of reinnervation occurs over a period of time [21]. Isolation of islets for grafting severs extrinsic nerves from the ganglia in the exocrine pancreas, leading to islet denervation, but an increase in sympathetic reinnervation occurs after 1 week [22]. For hair transplant surgery, follicular units are harvested either by dissecting the scalp slivers into follicular grafts or by using circular punches with a diameter of less than 1 mm [23]. This possibly interrupts the postganglionic sympathetic nerve fibers in the follicles, causing follicle denervation. On the other hand, no study has investigated the sympathetic innervation of transplanted follicles. Sato et al. dissected hair follicular units from the dorsal skin of C57BL/6-TgN (act-EGFP)OsbC14-Y01-FM131 (EGFP) mice, transplanted hair follicular units into the back skin of BALB/c-nu/nu mice, and reported that bulge regions from transplanted intact follicles form proper connections with the surrounding host nerve fibers; however, they did not show whether the nerve fibers are sympathetic nerves [24]. Vibrissa follicles of rodent animals possess a remarkable regenerative capacity and are frequently used to study the induction and regeneration of HFs [25,26,27]. Whisker follicles of rats are larger than pelage follicles and are arranged in horizontal and vertical rows. Therefore, rat vibrissa follicles are easily extracted and observed [25]. On the other hand, we found that mice with severe immune deficiencies (e.g., BALB/c-nu/nu mice or nu/nu mice) are commonly employed as recipients to prevent immunorejection and inflammation [7,24,27]. Therefore, here, we use vibrissa intact follicle xenograft BALB/c-nu/nu mouse models to explore the sympathetic reinnervation of xenografted follicles, which are also probed in upper follicle xenograft mouse models, to study the significance and potential applications of upper follicle grafts.

## 2. Materials and Methods

Ten-week-old female Sprague Dawley rats and six-week-old male BALB/c-nu/nu mice were purchased from Baishitong Biological Technology (Zhuhai, China) and Charles River (Beijing, China), respectively, and raised in a specific pathogen-free (SPF) environment. All animal care and experimental procedures were reviewed and approved by the Shantou University Medical College Ethics Committee (SUMC 2022-524).

Rats were euthanized by carbon dioxide asphyxiation. Their upper lip skin containing vibrissa follicles was cut out, and some were trimmed to approximately 7 mm × 1.3 mm × 1.3 mm tissue blocks before harvesting. Rat vibrissa follicular units were dissected from the remaining upper lip skin and the connective tissues surrounding the vibrissa follicles were carefully removed from the follicles using the procedures published by Iida et al., whose methods of recognizing anagen follicles morphologically were also applied [28]. Next, the anagen follicles were selected and hair shafts were cut as short as possible. The extracted follicles were divided into two groups: an intact follicle (IF) group with bulbs and an upper follicle (UF) group with bulbs excised. Some isolated follicles of each group were collected for histological examination and ELISAs (enzyme-linked immunosorbent assays), and the rest were stored in phosphate-buffered saline (PBS) at 4 °C and transplanted as quickly as possible. 

After recipient mice were anesthetized with pentobarbital sodium (50 mg/kg, intraperitoneally), small stab incisions were made with a needle (inner diameter 1.2 mm) in the dorsal skin and follicles were implanted. Then, mice were separated into two groups, an IF group and a UF group, according to the type of implanted follicle. 

Hair growth was observed and photographed, and the number of HFs with hair shafts grown out of the epidermis was counted on Day 1, Day 5 and Day 37 after transplantation. At 5 and 37 days, a subset of mice were anesthetized and transplanted follicles were harvested with a small amount of the surrounding skin. Subsequently, the samples were embedded in OCT, snap frozen, cut into 60 µm-thick sections and then fixed with 4% paraformaldehyde for 15 min, followed by 3 washes with PBS, for histological examination. The remaining mice were euthanized and transplants were collected. After flash freezing in liquid nitrogen, the samples were stored at −80 °C until ELISAs. The tissue blocks and extracted HFs, harvested as described above on Day 0, were treated using the same protocol.

Hematoxylin and eosin (HE) and immunofluorescence assay frozen sectioning were performed on the different groups of follicle tissues with roughly equal sizes along their median sagittal plane. HE staining was performed according to the standard protocols. Sixty-micrometer-thick sections were incubated for 2.5 min in hematoxylin and 4 min in eosin solutions. For immunofluorescence staining, slides were blocked with 5% donkey serum and 0.3% Triton X-100 in PBS for 2 h at room temperature and incubated with primary anti-TH antibody (rabbit, CST #58844, 1:100) overnight at 4 °C, followed by incubation with secondary Alexa Fluor 555-conjugated goat anti-rabbit IgG (L+H) (Beyotime A0453, 1:700) for 1.5 h at room temperature. Finally, samples were covered with an antifade mounting medium with DAPI (Beyotime P0131) and analyzed with a high-resolution confocal laser scanning microscope (Leica TCS SP8 STED) two or four hours later. A fibrous sheath of connective tissue, called the capsule, exists around the outer part of the vibrissa HF. Tyrosine hydroxylase staining was quantified to assess the sympathetic nerve density around the follicles, and the mean fluorescence intensity and areas within the capsule of vibrissa follicles were determined using Image J 1.53 software. 

Follicles were homogenized, and the norepinephrine concentration was determined using a commercial norepinephrine ELISA kit (Abnova KA1891) according to the manufacturer’s protocol. The norepinephrine concentration was calculated using a 4-parameter fit standard.

The data were presented using the medians or means ± standard deviation. The results were analyzed using SPSS 25.0 (IBM SPSS Statistics for Windows, Version 25.0). Variances of sympathetic nerve densities around the follicles and norepinephrine concentrations of follicles were evaluated using a two-way ANOVA, a one-way ANOVA or a Mann–Whitney test. * *p*  <  0.05; ** *p*  <  0.01; *** *p*  <  0.001; ns, not significant.

## 3. Results

### 3.1. Xenografts of Intact and Upper Follicles Can Enter Anagens

**Figure 1:**
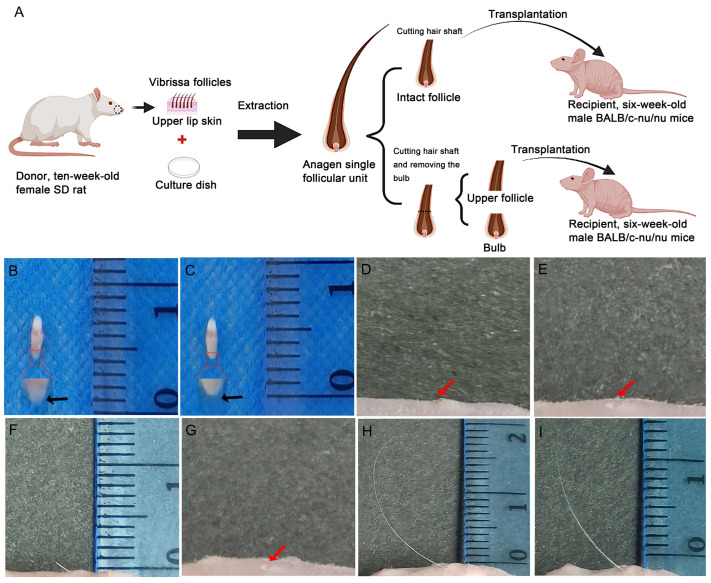
Experimental schematic of the SD rat vibrissa follicle xenograft model, macroscopic view and follow-up of the xenograft follicles in mice. (**A**) Experimental schematic of the SD rat vibrissa follicle xenograft model in BALB/c-nu/nu mice. (**B**–**I**) Macroscopic view and follow-up of the xenograft follicles in mice. Extracted intact follicle (IF) with a bulb (black arrow in **B**). Extracted upper follicle (UF) without a bulb (black arrow in **C**). Day 1 transplantation site wounds and skin elevations (red arrows in **D**,**E**) created by the transplanted follicles under the dorsal skin (IF group: **D**; UF group: **E**). The IF group on Day 5 (**F**). No hair growth (red arrow in **G**) was seen in the UF group on Day 5 after transplantation (**G**). Representative IF (**H**) and UF (**I**) hairs on Day 37.

Figure 1A shows our experimental scheme of rat vibrissa follicle xenografting into the dorsal skin of BALB/c-nu/nu mice. Gross specimens show the IF with a bulb (Figure 1B) and the UF without a bulb (Figure 1C) after extraction. To monitor hair growth after transplantation, the hair growth of the IF group and the UF group was observed after 1 day, 5 days and 37 days. No hair shaft outgrowth was observed 1 day after transplantation (IF group: Figure 1D; UF group: Figure 1E). Straight, thick, white hair shafts with a length of approximately 0.2 cm from the transplanted vibrissa follicles emerged from the epidermis on Day 5 in the IF group (Figure 1F), while the UF group remained hairless (Figure 1G). On Day 37, thick and white hair shafts with a cylindrical morphology and some degree of curvature had emerged in both groups (IF group: Figure 1H; UF group: Figure 1I), with the shaft length of the IF group (Figure 1H) being approximately 0.8 cm longer than hair shafts in the UF group (Figure 1I) at Day 37 and far more elongated than hair shafts on Day 5. A total of 25 IF follicles and 25 UF follicles were transplanted, and 14 hair shafts from the IF follicles and 8 hair shafts from the UF follicles emerged from the epidermis. The survival rates of xenografted IF follicles and UF follicles in our study were 56.00% and 32.00%, respectively.

**Figure 2:**
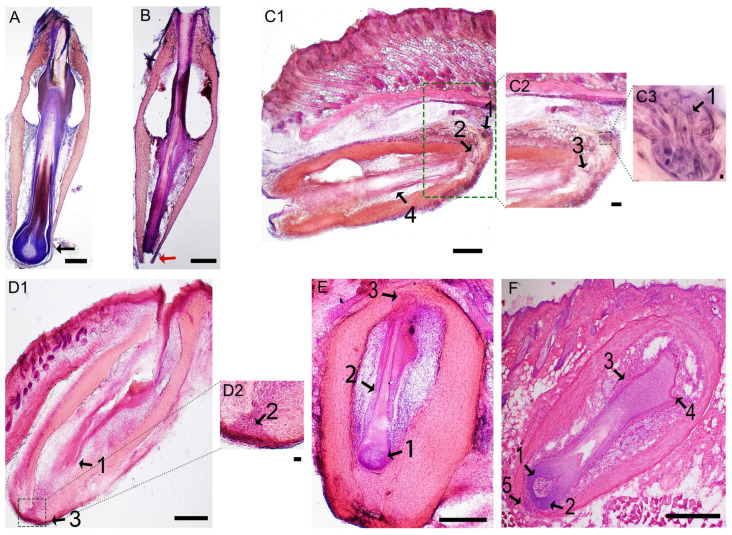
Xenografted intact and upper follicles can enter anagen. (**A**–**F**) Hematoxylin and eosin staining of IF and UF follicles at different time points. IF group with a bulb (black arrow in **A**) and UF group without a bulb (red arrow in **B**) on Day 0. The IF group, on Day 5 after transplantation (**C1**–**C3**), showing catagen V–VI and signs of a reduced compact ball-shaped DP (arrow 1 in **C1**,**C3**), epithelial constriction between germ capsule and dermal papilla (DP) (arrow 2 in **C1**), germ capsule formed by the outer root sheath (ORS) around the club hair (arrow 3 in **C2**) and obvious club hair appearance (arrow 4 in **C1**) [29]. The UF group on Day 5 after transplantation (**D1**,**D2**). The bottom ORS of the UF (arrow 1 in **D1**) was filled with dermal sheath (DS) cells (arrow 2 in **D2**) but the bulb had not yet formed (arrow 3 in **D1**). The IF group on Day 37 (**E**). Transplants had progressed to anagen V–VI and displayed a fully developed follicle with a large DP with a loose consistency (arrow 1 in **E**), a mature hair shaft with cuticle, cortex and medulla (arrow 2 in **E**) and the tip of hair shaft entering hair canal and emerging through the capsule (arrow 3 in **E**) [29]. The UF group on Day 37 (**F**) showing anagen III, characterized by a large DP with a loose consistency (arrow 1 in **F**), matrix cells forming the hair bulb (arrow 2 in **F**), a cone of keratinized cells appearing above the DP (arrow 3 in **F**) and the ripple-like appearance of the bulge (arrow 4 in **F**), but still with a narrow notch in the capsule below the newly formed bulb (arrow 5 in **F**) [29]. Scale bars, 160 μm (**C2**,**C3**,**D2**); 300 μm (elsewhere).

To study the morphological changes of transplanted follicles, microscopic examination was conducted by HE staining on Day 1, Day 5 and Day 37. HE staining showed an extracted IF on the anagen phase with a bulb (Figure 2A) and the UF without a bulb (Figure 2B). On Day 5 after transplantation, the IF under the skin entered catagen V–VI (mid-late dystrophic catagen) (Figure 2(C1–C3)). Figure 2(D1,D2) shows that on Day 5, the bottom ORS (outer root sheath) of the UF was filled with DS cells from the DS, but the bulb had not yet formed. On Day 37 after transplantation, both groups had progressed to anagen (IF group: Figure 2E; UF group: Figure 2F), with a regenerated bulb at the bottom in the UF group (Figure 2F). 

### 3.2. Partial Restoration of Sympathetic Reinnervation of the Transplanted Follicles

**Figure 3:**
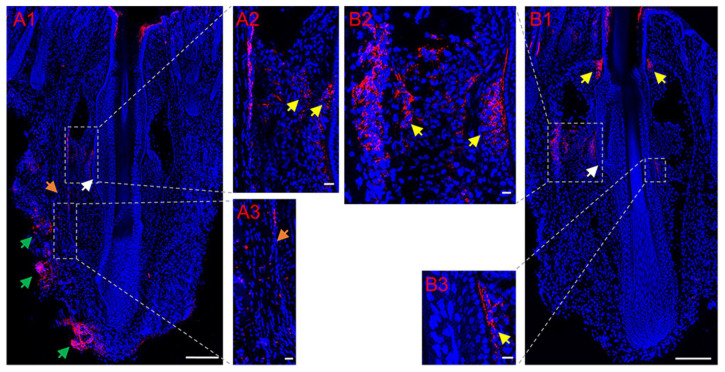
Expression of tyrosine hydroxylase (TH) around normal vibrissa follicles in situ using anti-TH (red) antibody. (**A**,**B**) TH-positive nerve fibers (yellow arrows in **A2** and **B1**–**B3**) originated from bundles (green arrows in **A1**) around the capsule (a fibrous sheath of connective tissue) (orange arrows in **A1**,**A3**) of vibrissa follicles, penetrated the capsule and innervated the bulge regions of hair follicle (HF) (white arrows in **A1**,**B1**) in situ. Scale bars, 160 μm (**A2**,**A3**,**B2**,**B3**); 300 μm (elsewhere).

**Figure 4:**
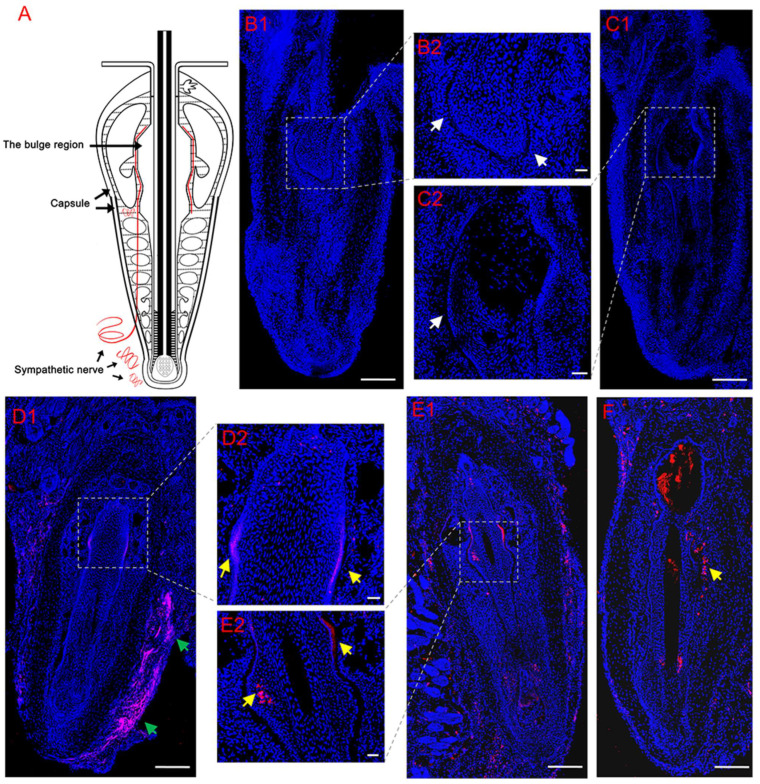
Temporal variation in changes in expression of TH around vibrissa xenografted follicles using anti-TH (red) antibody. (**A**) Schematic representation of sympathetic innervation of the vibrissa follicle in situ based on immunofluorescence observations. (**B**,**C**) Almost no TH-positive nerve fibers innervated the bulge regions of the HF (white arrows in **B2**,**C2**) in the IF (**B**) and UF (**C**) groups 5 days after transplantation. (**D**–**F**) By Day 37, small TH-positive nerve fibers (yellow arrows in **D2**,**E2**,**F**) reinnervated the bulge regions of the IF (**D**,**F**) and UF (**E**) groups, and the capsules of the IF group were hyperinnervated by TH-positive nerves (green arrow in **D1**). Scale bars, 160 μm (**B2**,**C2**,**D2**,**E2**); 300 μm (elsewhere).

**Figure 5:**
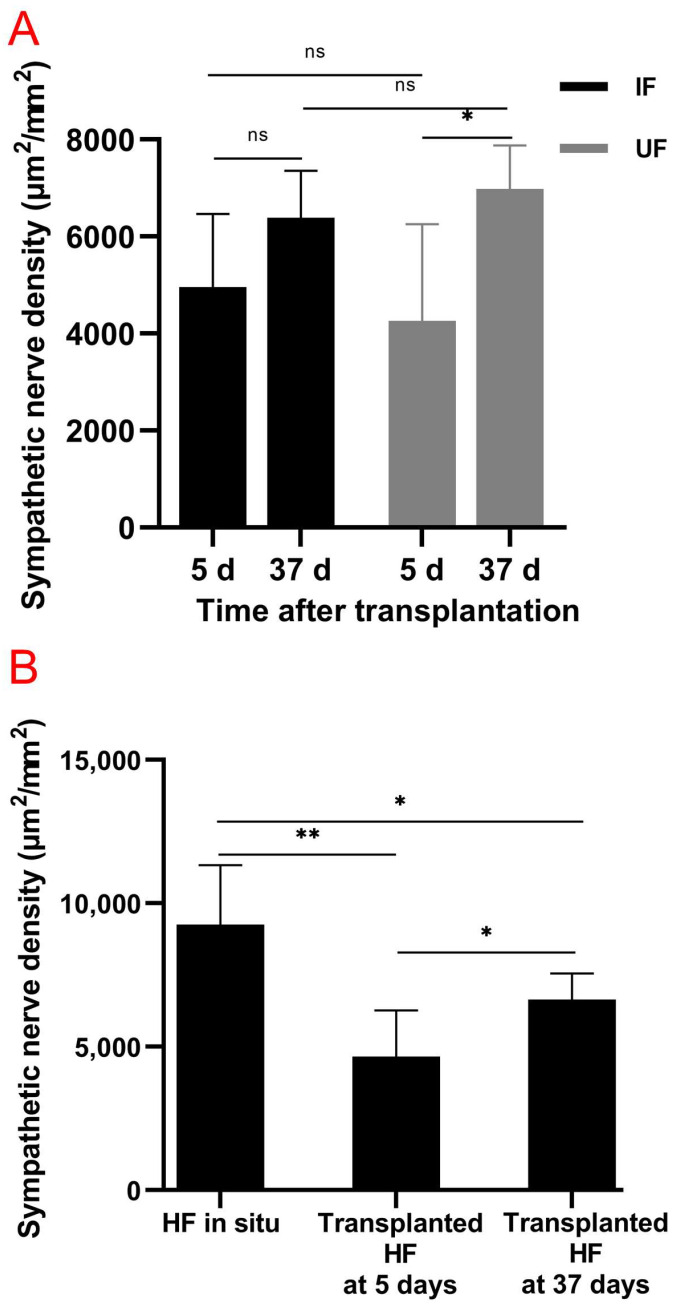
Partial restoration of sympathetic reinnervation around the transplanted follicles. (**A**) Change in sympathetic nerve density around the xenografted follicles in the IF and UF groups after 5 days and 37 days (two-way ANOVA, Bonferroni’s post test). (**B**) Change in sympathetic nerve density around normal vibrissa follicles in situ and xenografted follicles after 5 days and 37 days. Comparisons between xenografted follicles at 5 days and 37 days were conducted by a two-way ANOVA, while comparisons between normal vibrissa follicles in situ and xenografted follicles after 5 days or 37 days were performed using a one-way ANOVA. *n* = 3–10 mice for each condition. Bars represent means ± standard deviation. * *p*  <  0.05; ** *p*  <  0.01; ns, not significant.

Tyrosine hydroxylase is a marker for sympathetic nerves. In order to determine the temporal changes in TH around normal follicles in situ and transplanted follicles, immunofluorescence staining of TH was conducted. Immunofluorescence staining showed that the bulge regions of the HF were innervated by sympathetic nerve fibers (Figure 3(A1–A3), Figure 3(B1–B3)), which branched out from several TH-positive nerve fiber bundles around the capsules of normal vibrissa follicles in situ (Figure 3(A1)), penetrated the capsule (Figure 3(A1,A3)) and followed a path close to the ORS. Figure 4A shows a schematic representation of sympathetic innervation of the vibrissa follicle in situ. Almost no TH-positive nerves around the HFs were observed on Day 5 (IF group: Figure 4(B1,B2); UF group: Figure 4(C1,C2)), but Figure 4(D1,D2) (IF group) and Figure 4(E1,E2) (UF group) show that by Day 37, small, TH-positive nerve fibers reinnervated the bulge regions of HF, whose reinnervation by long and thick TH-positive nerve fibers was also found in the UF group (Figure 4F), but was not more than that around normal vibrissa follicles in situ. The capsules of HFs were hyperinnervated by TH-positive nerves, especially around the lower and middle sections of the capsules, and was mainly bundled on one side of the capsules (Figure 4(D1)). Quantitative data showed that the density of sympathetic nerves positive for TH significantly increased (1.64-fold, *p* = 0.037) after 37 days compared with that after 5 days in the UF group (Figure 5A). Figure 5B showed a significantly higher sympathetic nerve density around normal follicles in situ than those at 5 days and 37 days (1.99-fold, *p* = 0.003; 1.39-fold, *p* = 0.016, respectively) and a 1.45-fold increase in sympathetic nerve density in the xenografted follicles on Day 37 vs. that on Day 5 (*p* = 0.020).

**Figure 6:**
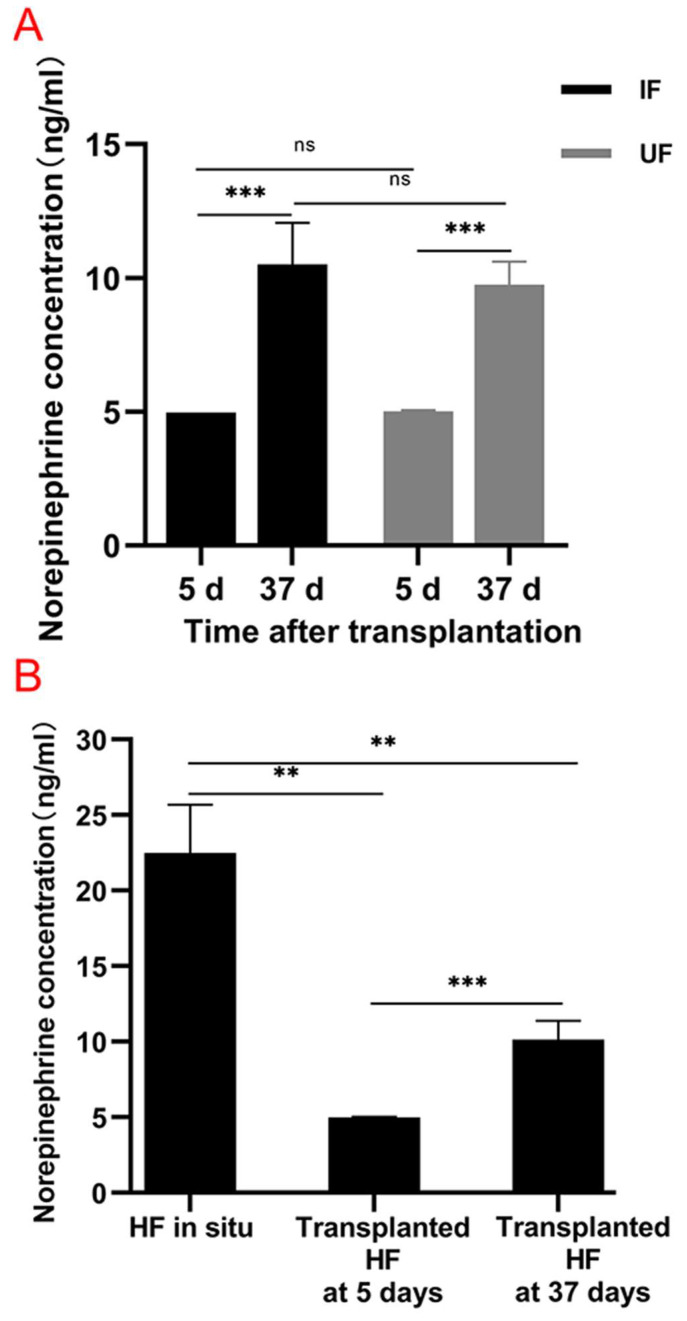
Partial restoration of norepinephrine concentration of the transplanted follicles. (**A**) Change in norepinephrine concentrations in the IF and UF groups after 5 days and 37 days (two-way ANOVA, Bonferroni’s post test). (**B**) Change in norepinephrine concentrations of normal vibrissa follicles in situ and xenografted follicles after 5 days and 37 days. Comparisons between xenografted follicles at 5 days and 37 days were conducted by a two-way ANOVA, while comparisons between normal vibrissa follicles in situ and xenografted follicles after 5 days or 37 days were performed using a Mann–Whitney test. *n* = 3–10 mice for each condition. Bars represent means ± standard deviation. ** *p*  <  0.01; *** *p*  <  0.001; ns, not significant. IF: intact follicle; UF: upper follicle; HF, hair follicle.

To investigate the changes in norepinephrine concentrations of normal follicles in situ and xenografted follicles after 5 days and 37 days, an ELISA was performed. Figure 6A shows that in the IF and UF groups, norepinephrine concentrations after 37 days were significantly elevated compared with those after 5 days (2.11-fold, *p* = 0.000; 1.94-fold, *p* = 0.000, respectively). Figure 6B shows that the norepinephrine concentration of normal follicles in situ was significantly higher than those after 5 days and 37 days (4.04-fold, *p* = 0.002; 2.04-fold, *p* = 0.002, respectively). A higher norepinephrine concentration was observed in the xenografted follicles on Day 37 compared with that on Day 5 (2.03-fold, *p* = 0.000). 

## 4. Discussion

Clinically, a significant portion of patients display a 1- to 3-month lag time for hair regrowth after hair transplantation, and follicles re-enter anagen six months later after hair transplantation to satisfactorily restore hair [30,31]. Similarly, we show that intact follicles enter dystrophic catagen 5 days after transplantation. This implies that after undergoing a traumatic transplantation and a large change in the microenvironment (such as denervation and loss of blood supply), intact follicles reset to a telogen effluvium-like state (an increased proportion of follicles entering the resting stage, leading to excessive shedding of hair clubs), just like that which occurs in HFs after chemotherapy, and then transition to telogen to launch a new hair cycle [4,32,33]. The early temporary hair loss of hair-grafted patients roughly corresponds to dystrophic catagen, when the club shaft is easily lost, in our mouse model [33]. In addition, upper follicles could enter anagen with regenerating bulbs after 37 days, as previous studies have shown [6,7,25]. Horne et al.suggested a new DP comes from cells at the bottom of the DS, consistent with our observation of DS cells occupying the bottom of the ORS of the upper follicle, where a new DP would be formed [6]. The upper follicles are consequently induced to enter into anagen because of the inductive capacity of the new DP [5]. This is indicative of survival of the transplanted upper follicle and the success of transplantation. However, our results are not in accordance with those of Wolbach et al., Crounse et al. and Geary et al., who destroyed or removed the DP and found that hair shaft ceased to grow [34,35,36]. In Rompolas et al.’s study, the progeny of HFSCs, which are located adjacent to the DP and are vulnerable to damage when the DP in telogen HFs is destroyed or removed, were ablated by laser, keeping the DP intact, and a similar growth rate to the surrounding undamaged HFs was shown, which contrasts with the finding that the HFs remained in a telogen phase after DP ablation [37]. It is likely that the main reason for the results of former three studies is that the researchers used experimental methods which do not show good selectivity for the destruction or removal of the DP and which may cause damage to HFSCs, which activate and differentiate into transit-amplifying cells (TACs) to form the hair matrix within the bulb in anagen HFs but not in telogen HFs [38]. Therefore, the removal of the progeny of HFSCs in telogen HFs may not affect anagen entry of HFs in Rompolas et al.’s study. This suggests that the intact upper segment of anagen HFs (except the DP) plays an essential role in restoration of a new bulb. 

We found that catagen follicles suffer from early denervation following follicle transplantation. Similar examples include cardiovascular regulatory changes in early heart transplant recipients, such as a lower cardiac index and heart rate variability, and denervation following lung reimplantation, which alters mucus secretion, delays clearance of mucus and reduces airway responsiveness to a variety of physiologic stimuli [21,39]. Sympathetic reinnervations similarly occur after some time. As for follicles, we found that the anagen follicles are reinnervated by regenerated small sympathetic nerve fibers 37 days after transplantation and that the norepinephrine concentration in follicles is reduced on Day 5 but increased on Day 37, but both the number of sympathetic nerve fibers and the norepinephrine concentration cannot return to normal. The thickness of the rat vibrissa follicle limits the observation of the whole process of sympathetic nerve fibers passing through the capsule to innervate the transplanted follicle by laser scanning confocal microscopy, even with longer observation times after follicle transplantation; additional experimental techniques such as lightsheet microscopy are required to overcome this limitation. The reason for anagen entry into transplanted follicles is most likely that the local release of norepinephrine after sympathetic reinnervation activates HFSCs [9,11]. Our study may help to understand the pathological mechanism of early hair loss in hair-grafted patients from the perspective of sympathetic nerves. In addition, a number of studies have provided morphological evidence of the sympathetic regrowth of transplanted organs, such as transplantations of pancreatic islets, the heart and the small bowel, where the time of sympathetic fiber regeneration ranges from 1 weeks to 15 years [16,21,22]. The recovery extent of sympathetic nerve fibers depends upon the individuals, organ type, follow-up time, implantation site, cellular composition of the transplant and donor animal type [16,21,22,40,41]. Taking our immunofluorescence staining and ELISA results together, we demonstrate that transplanted follicles suffer from sympathetic denervation immediately after hair transplantation, and sympathetic reinnervations around transplanted follicles occur later. Similarly, many studies reported reduced norepinephrine or catecholamine levels early after organ transplantation, especially within 7 days, but these gradually increased with time after organ transplantation and even returned to normal [16,22,42,43,44,45]. A study investigating sympathetic nerve restoration utilizing a catecholamine analog found that a young age, a fast and uncomplicated surgery and a low rejection frequency are more likely with reinnervation [46]. However, methods to induce early sympathetic reinnervation following organ transplantation are still an important issue that remains to be addressed. 

Sympathetic hyperinnervation of the capsule of transplanted intact follicles was observed in our study, in keeping with Olson et al.’s results of the over-expression of innervation in the iris after engraftment of a second iris into the anterior chamber and Buckley’s discovery of excessive sympathetic innervation of wounds in ear punch injuries of C57BL/6 mice [47,48]. This may be a result of excessive secretion of chemotactic factors such as the nerve growth factor from capsule or some compensatory mechanism [49]. Inaddition, similar to Mattson’s and Drake’s findings in vibrissa follicles of other animals, we showed that TH-positive nerve fibers penetrate the capsule and follow a path close to the bulge region of rat vibrassa follicles, and this seems a common characteristic of vibrissa follicles [50,51]. Furthermore, in our study, we observed that the bulge was reinnervated by regenerated sympathetic nerve fibers in both transplanted follicles, which suggests to us that some potential mechanism of axon guidance in the upper fragment of follicle may be in operation until the sympathetic nerve axons grow and innervate the bulge. Further studies are required to elucidate such mechanisms.

## 5. Conclusions

To conclude, we demonstrate the survival of intact and upper follicle xenografts and the partial restoration of sympathetic reinnervations of both transplanted follicles. Since sympathetic nerves contribute to hair growth, our study may help to understand the pathological mechanism of early hair loss in hair-grafted patients from the perspective of sympathetic nerves.

## Data Availability

The data presented in this study are available on request from the corresponding author.
